# The Screening of Anticholinergic Accumulation by Traditional Chinese Medicine

**DOI:** 10.3390/ijms19010018

**Published:** 2017-12-21

**Authors:** Ming Zhang, Misha Vrolijk, Guido R. M. M. Haenen

**Affiliations:** Department of Pharmacology and Toxicology, Maastricht University, 6200 MD Maastricht, The Netherlands; z.ming@maastrichtuniversity.nl (M.Z.); m.vrolijk@maastrichtuniversity.nl (M.V.)

**Keywords:** Traditional Chinese Medicine, anticholinergic accumulation, Total Atropine Equivalents

## Abstract

Many Western drugs can give rise to serious side effects due to their ability to bind to acetylcholine receptors in the brain. This aggravates when they are combined, which is known as anticholinergic accumulation (AA). Some bioactives in Traditional Chinese Medicine (TCM) are known to block acetylcholine receptors and thus potentially cause AA. The AA of TCM was screened by quantifying the displacement of [^3^H] pirenzepine on acetylcholine receptors in a rat brain homogenate. We used a new unit to express AA, namely the Total Atropine Equivalents (TOAT). The TOAT of various herbs used in TCM was very diverse and even negative for some herbs. This is indicative for the broadness of the pallet of ingredients used in TCM. Three TCM formulas were screened for AA: Ma Huang Decotion (MHD), Antiasthma Simplified Herbal Medicine intervention (ASHMI), and Yu Ping Feng San (YPFS). The TOAT of ASHMI was indicative for an additive effect of herbs used in it. Nevertheless, it can be calculated that one dose of ASHMI is probably too low to cause AA. The TOAT of YPFS was practically zero. This points to a protective interaction of AA. Remarkably, MHD gave a negative TOAT, indicating that the binding to the acetylcholine receptors was increased, which also circumvents AA. In conclusion, our results indicate that TCM is not prone to give AA and support that there is an intricate interaction between the various bioactives in TCM to cure diseases with minimal side effects.

## 1. Introduction

Acetylcholine receptors (AChRs), or cholinergic receptors, are important targets in pharmacotherapy. They are located in parasympathetic and sympathetic ganglia and in the brain. The muscarinic AChRs are G-protein coupled receptors and mediate a relatively slow response via second messenger cascades, while the nicotinic AChRs are ligand-gated ion channels that mediate a relatively fast response [1,2]. 

Many drugs have been designed to influence cholinergic transmission to obtain their therapeutic effects [3,4]. In addition, numerous drugs, as well as supplements, cause side effects by blocking AChRs [5]. When compounds that block AChRs are taken simultaneously, these side effects will accumulate. This interaction is known as anticholinergic accumulation (AA). AA may lead to dry mouth, blurred vison, falls, urinary disorders, impulsive behavior, and even cognitive impairment and delirium [6,7,8]. This is especially relevant for the elderly due to polypharmacy, changes in brain neurochemistry, and changes in pharmacokinetics and pharmacodynamics in this frail group [9,10,11]. 

Traditional Chinese Medicine (TCM) is increasingly valued in the Western world because of its effectiveness to treat diseases and its relatively mild side effects [12]. TCM is a dedicated combination of several herbs that has been developed over centuries, according to the “*Jun-Chen-Zuo-Shi*” principle. Within the combination, the *Jun* herb, main herb, is assisted by other herbs to stimulate the main pharmacological effect and to moderate side effects as well [13].

Some TCM contain bioactives that have anticholinergic activity [14]. The combination of these bioactives might aggravate this effect and cause AA. Nevertheless, TCM when used properly only results in mild side effects. This is in line with the dedicated combination of herbs in TCM. Recent studies aimed to decipher this combination principle confirmed that the interplay of the bioactives in the TCM circumvents side effects. For Western medication and supplements, the occurrence of AA is established [15]. However, there are hardly any data on the AA of TCM. This study is aimed to evaluate the potential occurrence of AA caused by TCM and to find out if AA is indeed circumvented by the dedicated combination.

To screen AA, in vitro receptor binding studies are performed. For a specific Western drug, the anticholinergic binding can be reflected by the dissociation equilibrium constant, K_D_ [16,17]. However, TCM is a mixture of compounds, and therefore it is impossible to express the anticholinergic activity of TCM by a K_D_. We propose a new unit, Total Atropine Equivalents (TOAT), to estimate and express the binding of TCM toward muscarinic AChRs on the rat brain membrane. The TOAT of an herb extract is defined as the concentration of atropine that gives a binding to the muscarinic AChRs equal to that of the herbal extract. With TOAT, the AA of herbs and the combination of herbs can be accurately expressed and predicted.

## 2. Results

**Figure 1:**
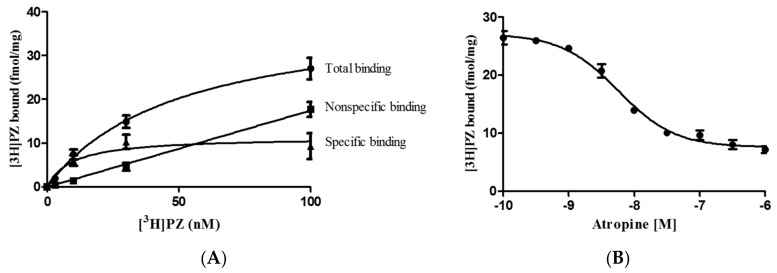
(**A**) Total, nonspecific and specific binding of [^3^H] pirenzepine ([^3^H]PZ) to muscarinic acetylcholine receptors (AChRs) in the rat brain homogenate. For the nonspecific binding, 10^−6^ M atropine was added to displace [^3^H]PZ from the AChRs. The K_P_ of [^3^H]PZ found was 9.3 × 10^−9^ M; (**B**) Displacement of [^3^H]PZ (30 nM) to muscarinic AChRs by atropine. The K_D_ of atropine found was 1.4 × 10^−9^ M. Data are shown as mean ± standard error of the mean (S.E.M.) (*n* = 3).

In Figure 1, The binding of [^3^H] pirenzepine ([^3^H]PZ) to muscarinic AChRs in rat brains is depicted. Non-specific binding was determined by incubating brain homogenate with a 100 µM atropine and various concentrations of [^3^H]PZ over the range 1 to 100 nM. The K_P_ of the binding of [^3^H]PZ to the AChRs was 9.3 × 10^−9^ M, and the concentration of muscarinic AChRs (B_max_) in the rat brain was 18.0 ± 1.3 fmol/mg protein (Figure 1A). A K_D_ of atropine for the AChRs found in the displacement experiment was 1.4 × 10^−9^ M (Figure 1B).

**Figure 2:**
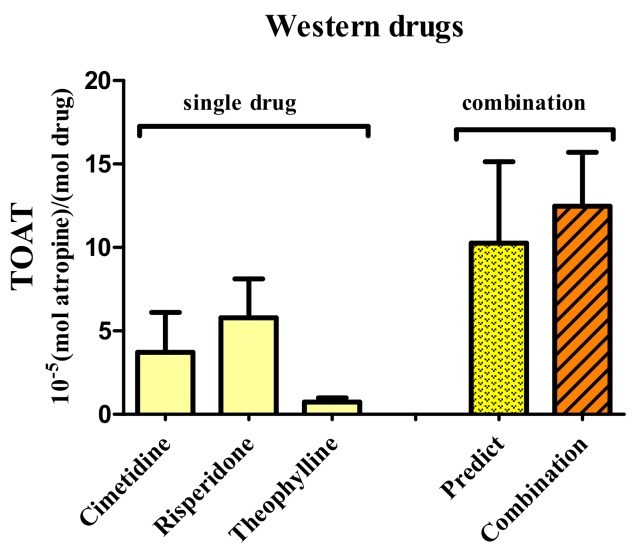
The Total Atropine Equivalents (TOAT) of the Western drugs cimetidine, risperidone, and theophylline compared to the TOAT of the combination of these Western drugs. The TOAT calculated by summing the TOAT of the single drugs was similar to the experimental TOAT. This indicated that the effects are additive.

To determine the binding of Western drugs and TCM to muscarinic AChRs, in vitro receptor binding studies are used. The TOAT of cimetidine, risperidone and theophylline were 4, 6 and 1 × 10^−5^ mol atropine/mol drug, respectively. The TOAT of the combination found experimentally was 11 × 10^−5^ mol atropine/mol drug, which is equal to the sum of the TOAT of the individual drugs (Figure 2). That the experimental TOAT equaled to the calculated TOAT, indicates that the effects of these Western drugs are additive and prone to cause AA.

**Figure 3:**
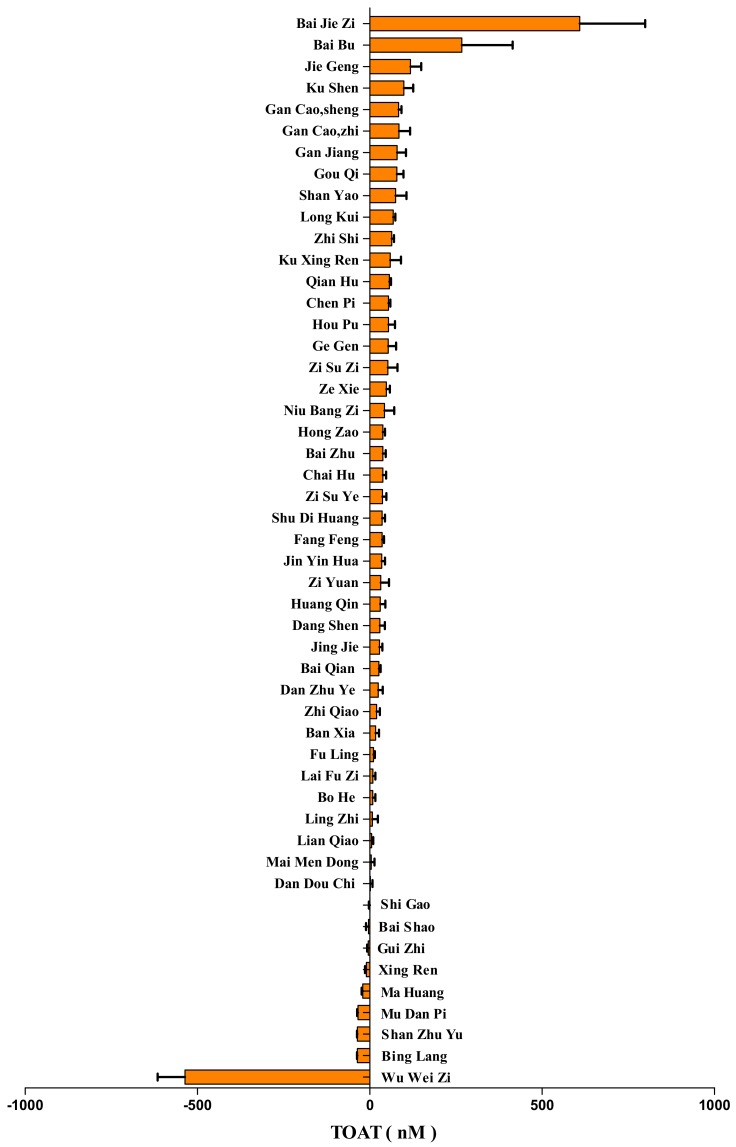
The TOAT of herbs used in Traditional Chinese Medicine (TCM). The TOAT of 50 herbs used in TCM. Data are shown as mean ± S.E.M. (*n* = 3).

Fifty different herbs used in TCM were screened for the binding to muscarinic AChRs and expressed as the TOAT (Figure 3). A high TOAT indicates that this herb can displace [^3^H]PZ effectively, whereas a low TOAT indicates relatively less displacement. The values of TOAT covered a wide range. Interestingly, some herbs had a negative TOAT, which means that these herbs increase the binding of [^3^H]PZ.

**Figure 4:**
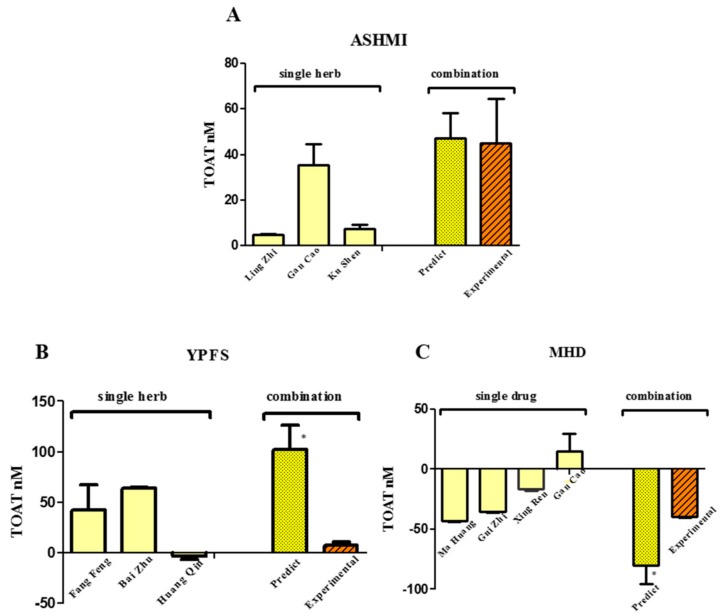
The TOAT of three different TCM formulas, and the TOAT of herbs used in the formulas. (**A**) Ma Huang Decotion (MHD); (**B**) Antiasthma Simplified Herbal Medicine intervention (ASHMI); (**C**) Yu Ping Feng San (YPFS). The predicted TOAT was calculated by summing the TOATs of the herbs. Predicted TOAT significantly different from the experimental TOAT (* *p* < 0.05).

TCM usually consists of a mixture of herbs. The TOATs of the TCM formulas and that of the individual herbs combined in the TCM formulas were determined (Figure 4). Similar to the combination of Western drugs, the experimental TOAT value of the TCM formula Antiasthma Simplified Herbal Medicine intervention (ASHMI), is equal to the sum of the TOAT of the individual herbs. That the experimental TOAT equaled the calculated TOAT indicates that the effects of the herbs in ASHMI are additive. In the formula Yu Ping Feng San (YPFS), the experimental TOAT is much lower than the calculated TOAT, which indicates that the blocking to receptors is decreased by using this combination. This would protect against AA. Remarkably, the formula Ma Huang Decotion (MHD) gives a negative TOAT, indicating that the binding to muscarinic AChRs was increased. This also points to a protection against AA by TCM.

**Figure 5:**
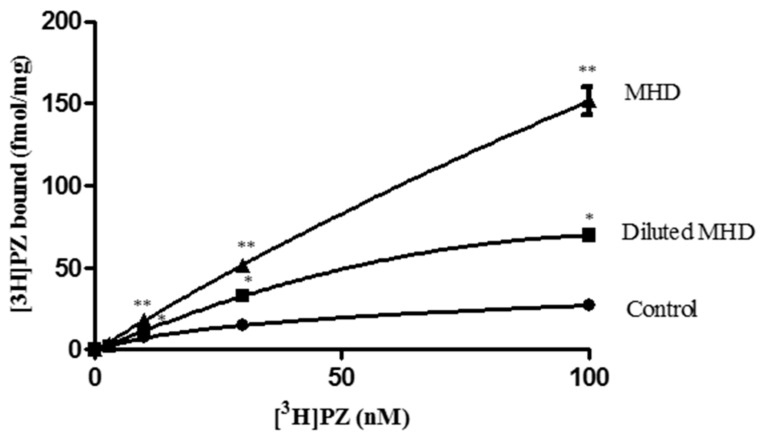
Total binding of [^3^H]PZ to muscarinic AChRs without and with 30 µL MHD, and 30 µL of 1:5 diluted MHD. MHD dose independently increase the binding of [^3^H]PZ. ** Significantly different from control and MHD. * Significantly different from control and diluted MHD.

To further investigate the increase in [^3^H]PZ binding by MHD, we test the effect of MHD on binding of various concentration of [^3^H]PZ to muscarinic AChRs. It was found that addition of MHD dose dependently increased the binding of [^3^H]PZ to AChRs over the entire concentration range tested (Figure 5).

## 3. Discussion

Compared with the relatively young Western drugs, TCM has matured over centuries. In TCM, several herbs are combined to treat a disease. When used properly, the side effects of TCM are relatively mild which is attributed to the intricate interplay of the various bioactives in the delicately assembled combination of herbs according to the “*Jun-Chen-Zuo-Shi*” principle [13]. This absence of major side effects is one of the reasons that TCM is increasingly valued in the Western societies. With regard to anticholinergic side effects, it should be noted that some herbs used in TCM are expected to display anticholinergic activity since they contain bioactives that are known to block AChRs [18]. The combination of these herbs, as is done in TCM, might even lead to AA. The aim of the present study was to determine the binding of TCM to AChRs to screen the potential risk of AA of TCM.

AA was screened in a receptor binding assay. We used a new unit, TOAT, to estimate and express the binding of TCM towards muscarinic AChRs. The TOAT is defined as the concentration of atropine that gives an equal displacement as that of an herbal extract. A relatively high TOAT means a higher concentration of atropine and is predictive for an increased risk of anticholinergic adverse effects. The TOAT can also be used to evaluate the interaction between different herbs.

The TOATs of cimetidine, risperidone, and theophylline were relatively small, and the combination of these three drugs results in a substantial TOAT value that was equal to the sum of individual TOATs of the drugs. This is indicative for an additive interaction of these drugs and for the potential occurrence of AA with their combination. Similar results were reported by Vrolijk et al., in which some drugs or supplements showed an additive interaction for AA when they were combined [15].

We screened 50 different herbs used in TCM for their anticholinergic activity. It appeared that the potency to displace the radiolabel from the AChRs greatly differed between the herb extracts. Some showed a relatively high TOAT value, indicative of their potency to block AChRs. Interestingly, some herb extracts had a negative TOAT value, meaning that the bioactives in these herbs increase the binding of the radiolabel to the AChRs. This high variation of the TOAT between the herb extracts demonstrates the broadness of the pallet of bioactives used in TCM.

We also screened some TCM formulas and compared them to the individual herbs they consist of. In herb formula ASHMI, the experimental TOAT value is similar to the TOAT calculated by summing the TOAT values of the individual herbs. That the experimental TOAT equals the calculated TOAT suggests that the effects are additive and indicates a risk of AA of the combination of these herbs. However, it should be noted that despite the additive interaction between the herbs, the TOAT was still relatively low. It can be calculated that 400 mL (the normal dose) of ASHMI that has a TOAT of 40 nM, is equal to a dose of 0.005 mg atropine, which is much lower than the therapeutic dose of atropine (1–2 mg). This indicates the ASHMI is not likely to give AA. For comparison, using the TOAT of cimetidine, which is 4 × 10^−5^ mol atropine/mol cimetidine, a regular dose of 800 mg cimetidine equals 0.032 mg atropine. This is 6 times more than the 0.005 mg of atropine equivalents of ASHMI, indicating that the risk with ASHMI is even lower than the relatively low risk of AA with cimetidine alone. Apparently, the anticholinergic effect of the bioactives in ASHMI is too low to give rise to AA. This is in line with the concept that TCM has a “soft” effect and not a “hard” effect like Western drugs.

Interestingly, in the herb formula YPFS, the experimental TOAT value is less than the calculated TOAT value. This implies that the blocking of AChRs is less than expected, and this is indicative for a protection against AA by this formula. This reveals the intricate interaction between the bioactives in TCM that is used to cure diseases with minimal side effects. This corroborates the general accepted image of TCM, that when used properly, TCM does not give rise to serious side effects. However, clinical studies on the AA of TCM is lacking at this moment, so according to Western standards, this is not “evidence based”.

The most striking finding of our study is that the TOAT of some herbs and formula MHD was negative. This means that the binding of the radiolabel is increased by adding compounds that were expected to reduce the binding. This does not fit in the generally accepted theory on receptor binding. Nevertheless, several phenomena observed in Western pharmacology might explain the increased binding observed with TCM. One is that the 3D structure of the receptor protein is affected by the bioactives in the TCM. The bioactives might bind to sites that induce a conformational change of the binding site that promotes binding [19]. Moreover, receptors are known to dimerize, and this might also be affected. It has been proved that heterodimerization can increase or decrease ligand binding and change the cellular response to receptor activation. Dimerization of receptors can also increase ligand binding [20]. In addition, the microenvironment of the receptor might be affected by TCM. Lipid rafts and caveolae regulate receptor activity at all the stages of their lifecycle [21]. By changing the microenvironment, TCM might also modulate the binding characteristics of the receptors.

We also tested the total binding of [^3^H]PZ to muscarinic AChRs together with MHD, which has a negative TOAT. It was shown that the binding of [^3^H]PZ was greatly increased. The extraordinary effects of TCM in the relatively simple receptor binding assay show that we only partially understand the intricate interplay of the bioactives in TCM.

## 4. Materials and Methods

### 4.1. Chemicals

[^3^H]PZ (84 Ci/mmol) was obtained from Perkin Elmer (Boston, MA, USA). Atropine, pirenzepine, cimetidine, risperidone, and theophylline were obtained from Sigma-Aldrich (St. Louis, MO, USA) and dissolved in assay buffer (50 mM potassium phosphate buffer, pH 7.4).

### 4.2. Preparation of Herb Extracts

The Chinese herbs were supplied by Chinaturel Import & Export B.V. (Den Haag, The Netherlands). Seven mL of distilled water was added to 1 g of the herb mixture (unless otherwise noted). After 30 min at room temperature, the mixture was boiled for 1 h. The extract was centrifuged at 5000× *g* for 15 min. The supernatant was filtered and stored at −20 °C [22].

Fifty diffrent herbs used to treat asthma were selected for the possibility of anticholinergic activity, since they may contain compounds that bind to AChRs. Three frequently clinical used anti-asthma herb formulas were also screened based on the potency to cause AA. They are MHD, ASHMI, and YPFS [23,24,25]. In each formula, all the herbs were combined in a fixed weight ratio. For MHD, this is Ma Huang (*Herba ephedra*):Gui Zhi (*Ramulus cinnamomi*):Xing Ren (*Semen armeniacae amarum*):Gan Cao (*Radix glycyrrhizae*) = 3:2:2:1 [26]. For ASHMI, this is Ling Zhi (*Gannoderma lucidum*):Gan Cao (*Radix glycyrrhizae*):Ku Shen (*Sophora flavescens*) = 20:9:3 [27]. For YPFS, this is Fang Feng (*Radix saposhnikoviae*):Bai Zhu (*Rhizoma atractylodis macrocephalae*):Huang Qi (*Radix astragali*) = 1:2:2 [28].

### 4.3. Tissue Preparation

Male Wistar Kyoto (WKY) rats (200–250 g; Charles River, Maastricht, The Netherlands) were sacrificed by CO_2_ inhalation, and brains were rapidly removed and put into the assay buffer. The brains were obtained from untreated control animals of animal studies with other objectives. These studies were conducted according to the ethical guidelines (EEC Council directives 86/609) after the animal experiments were approved by the University Animal Ethical Committee. This use of rest materials from other studies is encouraged to reduce the number of experimental animals (2014-110, 15 January 2015). The homogenization of rat Brains were carried out by using a Potter tube. The Brain homogenate was first centrifuged at 500× *g* for 10 min to remove unwanted pieces. Then this homogenate was centrifuged at a higher speed at 20,000× *g* for 15 min. All the centrifugations are carried out at 4 °C to protect the protein and aimed to remove all the substances that may interfere with solubility and radioligand binding assay. The final precipitated material was resuspended in assay buffer and diluted to reach the concentration of 1 mg of protein per 0.25 mL. The final brain homogenate was stored at −20 °C.

### 4.4. Binding Assays

For the [^3^H]PZ binding assay, the brain homogenate was used in a concentration given above. Analysis of [^3^H]PZ binding (final radioactive concentration: 100 µCi/mL) was determined as described by Michel et al. [29]. For saturation binding experiments, tissues were incubated in the assay buffer containing the radioligand and Chinese medicine extracts as indicated. The final assay volumes were 0.25 mL. The binding reactions were performed at 37 °C for 60 min and started by addition tissues to the mixture. After 1 h, 5 mL of ice-cold assay buffer was added, followed by rapid filtration through Whatman Grade GF/C filters. Each filter was washed with an additional 2–3 mL ice-cold buffer. Finally, the radioactivity of the counted filters was quantified using liquid scintillation counter in which the β emitters (^3^H) can be counted very effectively [15]. The amount of radioligand bound was less than 10% of total added ligand in all the experiments.

For saturation binding experiments, tissues were incubated for 60 min in a concentration of [^3^H]PZ (0–100 nM). Nonspecific binding was determined in parallel incubations using 1 µM atropine. Specific binding was calculated by subtracting nonspecific binding from the total binding. The affinity of atropine to muscarinic AChRs was detected after incubation of a fixed concentration of [^3^H]PZ (30 nM) and different concentrations of atropine (0.1 Nm–1 µM). In the displacement experiments, tissues were carried out in the presence of 30 nM of [^3^H]PZ and 30 µL herb extracts or 100 µM drugs in a total volume of 250 µL. Within one hour, [^3^H]PZ binding on muscarinic AChRs in the membrane of rat brain has reached an equilibrium. Fifty kinds of herb extracts were screened for binding to muscarinic AChRs. Moreover, herb formulas ASHMI, YPFS, and MHD were also screened for binding to muscarinic AChRs and a combination of drugs. The effect of formula MHD on the saturation binding of [^3^H]PZ to muscarinic AChRs was also determined. All binding experiments were performed at 37 °C. A control experiment showed that incubation of MHD with [^3^H]PZ (without the brain homogenate) did not result in extra [^3^H]PZ bound on the filter compounds to that of [^3^H]PZ alone.

The affinity of a specific compound to muscarinic AChRs can be expressed by the dissociation constant of that compound. The herbal extracts contain many compounds, including not yet identified compounds. It is not possible to describe the affinity of a mixture of compounds by the dissociation constant. Therefore, the concentration of a reference compound (i.e., atropine) that gives a displacement that is similar to that of the herb extract is calculated. This approach is comparable to that of the Trolox Equivalent Antioxidant Capacity (TEAC). The TEAC of a mixture (e.g., a food extract or a biological sample) gives the concentration of the reference compound Trolox that has an equal antioxidant capacity as that of the mixture [30,31]. Similar to the TEAC, the TOAT can be used to discriminate between synergistic and additive effects of the compounds in the mixture.

### 4.5. Calculation of the Total Atropine Equivalents (TOAT) for the Muscarinic Acetylcholine Receptors (AChRs)

**Figure 6:**
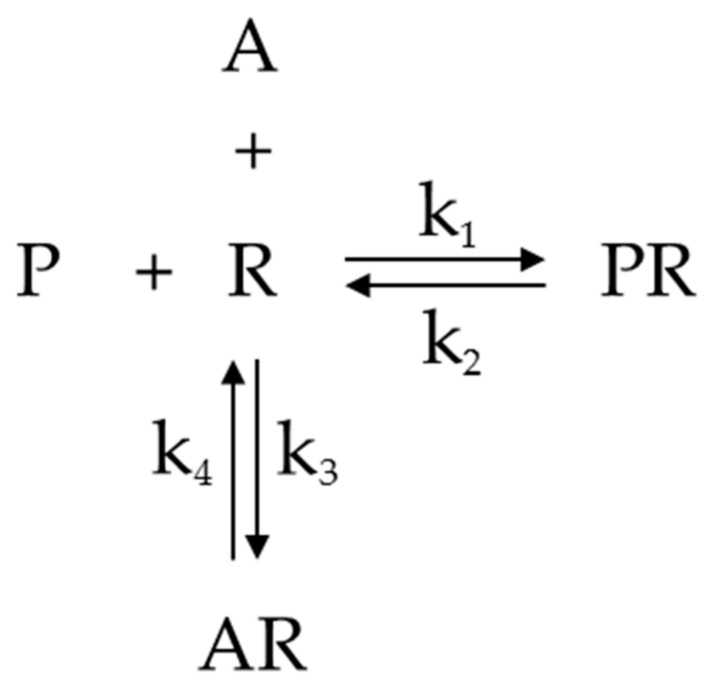
Competition of [^3^H]PZ (P) and atropine (A) for muscarinic AChRs (R). PR is P bond to R, and AR is A bond to R. k_1_ and k_2_ are rate constants for PR association and dissociation, respectively. k3 and k4 are rate constants for AR association and dissociation, respectively.

These calculations are based on the equilibrium depicted in Figure 6 [32,33].

In the equilibrium, P stands for [^3^H]PZ, R is the muscarinic AChRs, A is atropine. PR is P bond to R, and AR is A bond to R. k1 and k2 are rate constants for [^3^H]PZ-receptor association and dissociation, respectively. k_3_ and k_4_ are rate constants for atropine-receptor association and dissociation, respectively.

The equilibrium for binding of P to the receptor R can be given by the dissociation equilibrium constant K_P_, according to the following equation:

$$K_{P}\;=\;\frac{k_{2}}{k_{1}}=\frac{\left[P\right]*\left[R\right]}{\left[\mathrm{PR}\right]}$$

This equation can be transformed into Equation (1).

(1) $$[\mathrm{PR}]\;=\;\frac{\left[P\right]*\left[R\right]}{K_{P}}$$

Similarly, the equilibrium for binding of A to the receptor is given by the dissociation equilibrium constant K_D_ according to the following equation:

$$K_{D}\;=\;\frac{k_{4}}{k_{3}}\;=\;\frac{\left[A\right]*\left[R\right]}{\left[\mathrm{AR}\right]}$$

This equation can be transformed into Equation (2).

(2) $$[\mathrm{AR}]\;=\;\frac{\left[A\right]*\left[R\right]}{K_{D}}$$

In this assay, [^3^H]PZ is displaced by the herb extraction or drug. [A] is the concentration of atropine that has a similar displacement as herb extraction.

B is the concentration of receptors bound by [^3^H]PZ in the presence of herb extraction or drug, which is equal to [PR]. The total concentration of receptors, B_max_, is the sum of [R], [PR], and [AR].

$$B=\left[\mathrm{PR}\right]\;B_{\max}=\left[R\right]+\left[\mathrm{PR}\right]+\left[\mathrm{AR}\right]$$

It can be derived that the relative amount of receptors bound by [^3^H]PZ is

(3) $$\frac{B}{B_{\max}}\;=\;\frac{\left[\mathrm{PR}\right]}{\left[R]\;+\;[\mathrm{PR}]\;+\;[\mathrm{AR}\right]}$$

Substitution of Equations (1) and (2) into Equation (3) yields Equation (4).

(4) $$B\;=\;\frac{\left[\mathrm{PR}\right]*B_{\max}}{\left[R\right]\;+\;[\mathrm{PR}]\;+\;[\mathrm{AR}]}=\frac{\frac{\left[P\right]*\left[R\right]}{K_{P}}*B_{\max}}{\left[R\right]+\;\frac{\left[P\right]*\left[R\right]}{K_{P}}\;+\;\frac{\left[A\right]*\left[R\right]}{K_{D}}}=\frac{\left[\mathrm{AP}\right]*B_{\max}}{K_{P}\;+\;\left[P\right]+\;\frac{\left[A\right]*K_{P}}{K_{D}}}\;B\;=\;\frac{\left[P\right]*B_{\max}}{\left[P\right]\;+\;K_{P}\;(1\;+\;\frac{\left[A\right]}{K_{D}})}$$

The labelled [^3^H]PZ binds to the receptors and atropine competes with the labelled [^3^H]PZ for the receptors. Equation (4) can be rearranged into Equation (5).

(5) $$[A]=\;\frac{K_{D}\;*([P]\;*\;B_{\max}\;-\;[P]*B\;-\;K_{P}\;*\;B)}{K_{P}*B}$$

The formula to calculate the TOAT is

(6) $$\mathrm{TOAT}\;=\;[A]\;=\;\frac{K_{D}*([P]\;*\;B_{\max}\;-\;[P]*B\;-\;K_{P}\;*\;B)}{K_{D}*B}*D$$

In this equation, D is a factor of 8.33 that accounts for the dilution in the assay, in which 30 µL of herb extract was used in a total volume of 250 µL. To determine the TOAT, we used a K_D_ of atropine to muscarinic AChRs of 9.34 × 10^−9^ M and a K_P_ of [^3^H]PZ to muscarinic AChRs of 1.40 × 10^−9^ M. Both were determined experimentally and comparable to the value found in literature. The dimension of the TOAT of the herb extract determined according to this procedure is *nM*.

B_max_ was determined from the specific binding of [^3^H]PZ to the receptors without the tested compounds. The formula is

$$B_{\max}\;=\;\frac{B*(\left[P\right]\;+\;K_{P})}{\left[P\right]}$$

B* is the specific binding of [^3^H]PZ to the receptors without tested compounds.

The TOAT of a drug was determined by the displacement of [^3^H]PZ by a 100 µM solution of the drugs. The concentration of atropine that gave the same displacement as 100 µM (final concentration) of the drug was calculated according to Equation (6), with D = 1. The TOAT of the drug reflects the potency of drug to displace [^3^H]PZ relative to that of atropine on a molar basis. Therefore the dimension of the TOAT of a drug obtained in this way is 10^−5^ (mol atropine)/(mol drug).

### 4.6. Statistics

Data are shown as mean ± standard error of the mean (S.E.M.). *n* refers to the number of independent experiments that repeated. Data were analyzed in Prism 5 (GraphPad Software Inc., La Jolla, CA, USA). The data of total binding, nonspecific and specific binding were fitted to a nonlinear regression curve-one binding site. The kinetic parameters, K_D_ of [^3^H]PZ and B_max_, were calculated by fitting to one site-specific binding. The results of atropine displacement experiments were fitted to one site binding competitive (fitting K_i_) to calculated K_D_. Data were analyzed using a two-tailed Student’s *t*-test. *p*-value < 0.05 were considered statistically significant.

## 5. Conclusions

In conclusion, a radioligand binding assay interpreted using the TOAT seems a convenient way to screen for AA of TCM. Our results indicate that when used properly, TCM is not prone to give AA. The results also show that the effects of TCM on radioligand binding are complex and that contra intuitive interactions occur. This demonstrates the broadness of pallet of the bioactives as well as their intricate interplay in TCM, which is designed to cure diseases with minimal side effects. Western pharmacology might benefit from further unraveling of these interactions, which will also help to fully appreciate TCM in the West.

## Abbreviations

AA	Anticholinergic Accumulation
TCM	Traditional Chinese Medicine
TOAT	Total Atropine Equivalents
MHD	Ma Huang Decotion
ASHMI	Antiasthma Simplified Herbal Medicine Intervention
YPFS	Yu Ping Feng San
AChRs	Acetylcholine Receptors
[^3^H]PZ	[^3^H]pirenzepine
TEAC	Trolox Equivalent Antioxidant Capacity
